# Supplementary material for: Patterns of Intron Gain and Loss in Fungi
Source: PLoS Biol. 2004 Nov 30;2(12):e422. doi: 10.1371/journal.pbio.0020422 (PMC532390; doi:10.1371/journal.pbio.0020422)
Supplement: Table S1 — Also available at http://genes.mit.edu/NielsenEtAl/. (4.3 MB ZIP). [file pbio.0020422.st001.zip › NielsenEtAl/html/461.html]

AN4547.1.NCU01992.1.MG02634.1.FG08840.1


```
 CLUSTAL W (1.82) Multiple Sequence Alignments - Introns Inserted


Sequence 1: MG02634.1	917 aa
Sequence 2: FG08840.1	933 aa
Sequence 3: NCU01992.1	916 aa
Sequence 4: AN4547.1	912 aa
Alignment Length: 936 aa
Number Identitical Residues: 646 aa
Alignment Score (without introns) 27549


MG02634.1 	MNYGKKDEDAENGLVKVDRTQVFQEA1RLFNSSPIQPRQCRILLTKIALLLYTGEKFPQN
NCU01992.1	MNYGKKDEDAETGLVKVDRTQVFQEA1RLFNNSPIQPRKCRILLTKIALLLYTGERFPTN
FG08840.1 	MSYGKKDEDADLGLVKVDRTQVFQEA1RLFNSSPIQPRRCRILLTKIALLLYTGEKFPTN
AN4547.1  	MSYMKKDEDADQTMIKLDRTSVFQDA1RLFNSSPISPRQCRTLLTKIAVLVFTGEQFPTN
          	*.* ******:  ::*:***.***:* ****.***.**:** ******:*::***:** *

MG02634.1 	EATTLFFGISKLFQNKDASLRQMVHLVIKELANSAEDIIMVTSTIMKDTGGSTDIIFRPN
NCU01992.1	EATTLFFGISKLFQNKDASLRQMVHLVIKELANSAEDIIMVTSTIMKDTGGSTDAIYRPN
FG08840.1 	EATTLFFGISKLFQNKDASLRQMVHLVIKELANSAEDIIMVTSTIMKDTGGSTDTIYRPN
AN4547.1  	EATTLFFGISKLFQNKDPSLRQMVYLILKELANTAEDVIMSTSIIMKDTAVGSDVLYRAN
          	*****************.******:*::*****:***:** ** *****. .:* ::*.*

MG02634.1 	AIRALCRIID0ASTVQSIERVMKTAIVDKNPSVSSAALVSSYHLLPIARDVVRRWQSETQ
NCU01992.1	AIRALCRIID0ATTVQSIERVMKTAIVDRNPSVSSAALVSSYHLLPVAKDVVKRWQNETS
FG08840.1 	AIRALCRIID0ATTVQSIERVMKTAIVDKNPSVSSAALVSSYHLLPIAKDVVRRWQSETQ
AN4547.1  	AIRALCRIID0G-----IERLIKTAIVDKTPSVSSAALVSSYHLLPIARDVVRRWQSETQ
          	********** .     ***::******:.****************:*:***:***.**.

MG02634.1 	EAAASNKSSGGFSLGFSSASSSMPVNNS-TMSQYHAIGLLYQMRMHDRMALVKMVQQFGA
NCU01992.1	EAAANTKSTGGFSLGFGSSNRDLPINSS-TMTQYHAIGLLYQMRMHDRMALVKMVQQFGA
FG08840.1 	EAAASNKSSGGFSLGFSTSSSQVPMNHS-TMSQYHAVGLLYQMRMHDRMALVKMVQQFGA
AN4547.1  	EAASSSKQSTGF-LGFGGSSQAHAISQSNFMTQYHAIGLLYQMRSHDRMALVKMVQQYGA
          	***:..*.: ** ***. :.   .:. *. *:****:******* ************:**

MG02634.1 	PGAVKSPAALVMLVRLAAQLAEEDPQLRRPMMQLLDGWLRHKSEMVNFEAAKAICDMRDV
NCU01992.1	PGAVKNPAALMLLVRLAAQLADEDPHLRKPMMQLLDGWLRHKSEMVNFEAAKAICDMRDV
FG08840.1 	PGALKSPAAIVMLVRLAAQLAEEDASLRKPMMQLLDGWLRHKSEMVNFEAAKAICDMRDV
AN4547.1  	AGVVKSPAALVLLVRLAAKLAEEDSGLRKPMMQMLDGWLRHKHEMVNFEAAKAICDIRDV
          	.*.:*.***:::******:**:**. **:****:******** *************:***

MG02634.1 	TDAEVSQAVHVLQLFLTSPRAVTKFAALRILHNFASFKPNTVNVCNPDIELLISNSNRSI
NCU01992.1	TDAEVTQAVHVLQLFLTSPRAVTKFAALRILHNFASFKPAAVAVCNPDIELLISNSNRSI
FG08840.1 	TDAEVSQAVHVLQLFLTSPRAVTKFAALRILHNFASFKPNAVNPCNPDIELLISNSNRSI
AN4547.1  	SDAEASQAVHVLQLFLSSPRAITKFAAIRILHRFASFKPHVVNVCNPDIEALISNTNRSI
          	:***.:**********:****:*****:****.****** .*  ****** ****:****

MG02634.1 	ATFAITTLLKTGNEASVDRLMKQITGFMSEITDEFKITIVEAIRTLCLKFPSKQAGMLAF
NCU01992.1	ATFAITTLLKTGNEASVDRLMKQISGFMSEITDEFKITIVEAIRTLCLKFPSKQAGMLQF
FG08840.1 	ATFAITTLLKTGNEASVDRLMKQISTFMSEITDEFKITIVEAIRTLCLKFPSKQAGMLTF
AN4547.1  	ATFAITTLLKTGNEASVDRLMKQISGFMADITDEFKITIVEAIRTLCLKFPSKQAGMLSF
          	************************: **::**************************** *

MG02634.1 	LSGILRDEGGYEFKRAVVESMFDLIKFVPDSKEDA~LAHLCEFIEDCEFTKLAVRILHLL
NCU01992.1	LSGILRDEGGYEFKRAVVESMFDLIKFVPESKEEA~LAHLCEFIEDCEFTKLAVRVLHLL
FG08840.1 	LSGILRDEGGYEFKRAVVESMFDLIKFVPDSKEDA~LAHLCEFIEDCEFTKLAVRILHLI
AN4547.1  	LSGILRDEGGYEFKRSVVESMFDLIKFVPESKEDA1LAHLCEFIEDCEFTKLSVRVLHLL
          	***************:*************:***:* ****************:**:***:

MG02634.1 	GLEGPKTSQPTKYIRYIYNRVVLENAIVRAAAVTALAKFGVGQKDPEVKRSVHVLLTRCL
NCU01992.1	GLEGPKTSQPTKYIRYIYNRVVLENSIVRAAAVTALAKFGVGQKDPEVKRSVEVLLTRCL
FG08840.1 	GLEGPKTAQPTKYIRYIYNRVVLENAIVRAAAVTALAKFGVGQKDPDVKSSVRVLLTRCL
AN4547.1  	GVEGPKTSQPTKYIRYIYNRVVLENAIVRAAAVTALAKFGVGQKDPEVKSSVSVLLTRCL
          	*:*****:*****************:********************:** ** *******

MG02634.1 	DDVDDEVRDRAALNLRLMDEED-ALAEKFVKN-----------------D1SMFSLPYFE
NCU01992.1	DDVDDEVRDRAALNLKLMKEED-ELANRFVKN-----------------D1SMFSLPFFE
FG08840.1 	DDVDDEVRDRAALNLKLMNEEDDEMAVRFVKNGKKTITPHRQTVTNAATE~NMFSLPYFE
AN4547.1  	DDTDDEVRDRAALNLRLMAEED-EMANLFIKN-----------------E1SMYSLSTFE
          	**.************:** ***  :*  *:**                 : .*:**. **

MG02634.1 	HQLVMYVTSDDKSTFDSPFDISKIPVVTREQADAEDRTKKLTATTPSLKPPKVGPTKAAP
NCU01992.1	HQLVTYVTSDDRSTFEAPFDISKIPVVTREQADAEDRTKKLTATTPSLKPPKVGPTKAAP
FG08840.1 	QQLVMYVTSEEASAFDSPFDISKIPVVTREQADAEDRSKKLIATTPTLKAPKVGPTKAT-
AN4547.1  	HQLVMYVTSGDKETFATAFDVSTIPVVSQEQALAEERTKKLTSATPTLKAPSTGPPKGAK
          	:*** **** : .:* :.**:*.****::*** **:*:*** ::**:**.*..**.*.: 

MG02634.1 	TGAEAAASATAAAQKYAQELMQIPEMKEFGSVLKSSPIVELTEAETEYVVSVVKHIFKEH
NCU01992.1	TSAEAAASASAAAQKYAQELMQIPEMAEFGAVLKSSPVVELTEAETEYVVGVVKHIFKEH
FG08840.1 	-GAEAVATASAQAQKYAQELMEIPEMQEFGNVLKSSPLIELTEAETEYVVSLVKHIFKEH
AN4547.1  	ANGVAEAATAAATQKYAEQLMQIPELKAYGTLLKSSVPVELSESETEYVVTAVKHIFKEH
          	:.. * *:::* :****::**:***:  :* :****  :**:*:******  ********

MG02634.1 	IVLQFEVKNTLPSTVLENVSVVATPSDEEELEELFIIEAEKLPTDEPGKVYVAFKKVSGE
NCU01992.1	IVLQYEVKNTLPDTVLENVSVVATPA-EEELEELFIIQAEKLETDVPGKVYVAFRKVSGE
FG08840.1 	VVLQYEVKNTLPDTVLENVSVVATPADDEELEEVFIIQAEKLATDEPGKVYVAFKKIGGE
AN4547.1  	IVLQYDIKNTLPDTVLEDVTVVATPSEEDILEEEFIVPAPKLNTNEPGVVYVTFKKLEGE
          	:***:::*****.****:*:*****:.:: *** **: * ** *: ** ***:*:*: **

MG02634.1 	GSMPISTFSNSLKFTTKEIDPTTNEPEDEGYEDEYEVAEFDVSGSDYVIPTFASNFNHLW
NCU01992.1	GSLPVSTFSNVLKFTSKEIDPSTGEPEETGYDDEYEVAEFDLAGSDYVIPVFASNFAHIW
FG08840.1 	ASLPISTFTNVLKFTSKEIDPSTGEPEDTGYDDEYEVAEFDLSGSDYVIPTFAGNFSHIW
AN4547.1  	QSVPVVSFTNNLKFTTKEIDPTTGEPEDSGYEDEYQVNDLELTGSDYVIPTFAGSFDHVW
          	 *:*: :*:* ****:*****:*.***: **:***:* :::::*******.**..* *:*

MG02634.1 	EQVGAAGEEAEETLQLSAMKSIAE1ATEQLSKALSLQPLEGTDVPVNQTTHTLKLLGKTV
NCU01992.1	EQVGASGEEAEETLLLSSMKSISD1ATEQLAKTLSLQPLEGTDVPLNQTTHTLKLLGKTV
FG08840.1 	EQVGASGEEVTETLQLSGMESIAD1ATEQLTKALSLQPLEGTDVPVNQTTHTLKLLGKTV
AN4547.1  	EQAGANGEEVSETLQLSNLKGIAD1ATEQLISTLSLQPLEGTDVALSNSTHTLKLFGKTV
          	**.** ***. *** ** ::.*:: ***** .:***********.:.::******:****

MG02634.1 	NGGRVVANVRMAYSSKSGVTTKITVRSEEEGVAALVIASVE
NCU01992.1	NGGRVVANVRMAFSAKSGVTTKITVRSEEEGVAALIVASVA
FG08840.1 	GGGRVVANVRMAFSSKTGVTTKITVRSEEENVAALVIASVA
AN4547.1  	LGGRVAALIKMAYSTKTGVTTKITVRAEEGGVAPAVIASLA
          	 ****.* ::**:*:*:*********:** .**. ::**:
```
